# Hypomanic Switch Induced by Lurasidone: A Case Report

**DOI:** 10.1192/bjo.2025.10754

**Published:** 2025-06-20

**Authors:** Fatma Sena Ozbal, Baxi Sinha

**Affiliations:** 1 Tees, Esk and Wear Valleys NHS Foundation Trust, Middlesbrough, United Kingdom; 2 Tees, Esk and Wear Valleys NHS Foundation Trust, Stockton-On-Tees, United Kingdom

## Abstract

**Aims:** Bipolar Affective Disorder (BAD) is known to present with manic or hypomanic episodes, along with depressive episodes occurring at different times. Considerable within-normal-range mood variations between episodes are expected. In some cases, mania or hypomania can be induced by medications. While well-established medications such as corticosteroids and levodopa are known to induce mania, a careful examination of possible triggers for manic switches is essential to ensure individualized patient care.

**Methods:** This 34-year-old Caucasian female patient presented with depressive disorder. She has a history of multiple depressive episodes. She had been on sertraline for seven months for her most recent depressive episode. However, due to excessive sweating as a reported side effect, her medication was switched to fluoxetine at 20 mg daily, which was subsequently increased to 40 mg after three weeks. On the fifth day after the dose increase, she presented with hypomanic symptoms, changing her diagnosis to Bipolar Affective Disorder (BAD), current episode hypomania. Fluoxetine was identified as the causative agent for this switch. However, at the patient’s request, fluoxetine was continued with antipsychotic and/or mood stabilizer cover, as the patient retained the capacity to make decisions regarding her medical management. The patient was otherwise fit and well, with no recent drug or alcohol involvement. She was prescribed lorazepam 0.5 mg PRN QDS, zopiclone 7.5 mg PRN nightly, and risperidone 1 mg BD in addition to fluoxetine 40 mg daily. Due to drowsiness associated with risperidone, she was commenced on lithium PR titration. Two weeks later, her subsequent symptoms were consistent with mixed affective states. The fluoxetine was discontinued with the patient’s consent. Lurasidone 37 mg was initiated alongside lithium (Priadel XL) 600 mg. Four days after the medication change, the patient reported feeling sedated and experiencing low energy levels. After this review, her lithium dose was increased to 800 mg. Eight days after initiating lurasidone, the patient called the clinic with marked irritability. She reported having high energy levels and feeling very well. The review revealed another hypomanic switch with marked rapid speech and reduced need for sleep. The symptoms of hypomania have resolved following discontinuation of lurasidone.

**Results:** This case report highlights the possible hypomanic switch associated with the use of lurasidone. With several new drugs approved for mental illnesses including BAD, it is crucial to monitor their side-effect profiles to ensure safer and more effective management.

**Conclusion:** There are several potential confounders identified in this case.

